# Development and validation of near-infrared spectroscopy for the prediction of forage quality parameters in *Lolium multiflorum*

**DOI:** 10.7717/peerj.3867

**Published:** 2017-10-03

**Authors:** Zhongfu Yang, Gang Nie, Ling Pan, Yan Zhang, Linkai Huang, Xiao Ma, Xinquan Zhang

**Affiliations:** Department of Grassland Science, College of Animal Science and Technology, Sichuan Agricultural University, Chengdu, China

**Keywords:** *Lolium multiflorum*, Forage quality, Near-infrared spectroscopy, Multivariate calibration

## Abstract

Italian ryegrass (*Lolium multiflorum*) is an important cool-season, annual forage crop for the grassland rotation system in Southern China. The primary aim of breeding programs is always to seek to improve forage quality in the animal productivity system; however, it is time- and labor-consuming when analyzed excessive large number of samples. The main objectives of this study were to construct near-infrared reflectance spectroscopy (NIRS) models to predict the forage chemistry quality of Italian ryegrass including the concentrations of crude protein (CP), acid detergent fiber (ADF), neutral detergent fiber (NDF), and water soluble carbohydrate (WSC). The results showed that a broader range of CP, NDF, ADF and WSC contents (%DM) were obtained (4.45–30.60, 21.29–60.47, 11.66–36.17 and 3.95–51.52, respectively) from the samples selected for developing NIRS models. In addition, the critical wavelengths identified in this study to construct optimal NIRS models were located in 4,247–6,102 and 4,247–5,450 cm^-1^ for CP and NDF content, and both wavelengths 5,446–6,102 and 4,247–4,602 cm^-1^ could for ADF and WSC. Finally, the optimal models were developed based on the laboratory data and the spectral information by partial least squares (PLS) regression, with relatively high coefficients of determination (*R*^2^_CV_, CP = 0.99, NDF = 0.94, ADF = 0.92, WSC = 0.88), ratio of prediction to devitation (RPD, CP = 8.58, NDF = 4.25, ADF = 3.64, WSC = 3.10). The further statistics of prediction errors relative to laboratory (PRL) and the range error ratio (RER) give excellent assessments of the models with the PRL ratios lower than 2 and the RER values greater than 10. The NIRS models were validated using a completely independent set of samples and have coefficients of determination (*R*^2^_V_, CP = 0.99, NDF = 0.91, ADF = 0.95, WSC = 0.91) and ratio of prediction to deviation (RPD, CP = 9.37, NDF = 3.44, ADF = 4.40, WSC = 3.39). The result suggested that routine screening for forage quality parameters with large numbers of samples is available with the NIRS model in Italian ryegrass breeding programs, as well as facilitating graziers to monitor the forage development stage for improving grazing efficiency.

## Introduction

Italian ryegrass (*Lolium multiflorum*) is one of the most famous annual forage grasses, and is widely used for the cereal-forage rotation system in south China owing to its high productivity and palatability, excellent resprouting and easy plantability (Carámbula & Carámbula, 1977; MAIA, 1995). As green-feed or silage for livestock daily ration, the good forage quality of Italian ryegrass cultivar is the primary goal for famers and breeders (Casler & Vogel, 1999). The content of crude protein (CP), neutral detergent fiber (NDF) and acid detergent fiber (ADF), and water soluble carbohydrate (WSC) are the three most important forage quality parameters which determine the forage intake and digestibility for livestock (Mott & Moore, 1970). The high CP content increased the milk and milk protein yield (Mäntysaari et al., 2004) and the NDF, ADF are well correlated with digestibility for livestock animals (Agbagla-Dohnani et al., 2001; Agnihotri et al., 2003; Dutta, Sharma & Hasan, 1999; Suksombat, 2004; Yépez et al., 2004), as well as the WSC may improve the balance and synchrony of the nitrogen and carbon supply to the rumen (Miller et al., 2001). Furthermore, the appropriate content of the WSC could prevent clostridial from fermenting, which is a critical parameter for silage production (Haigh, 1990; Pettersson & Lindgren, 1990). Traditional methods for determining the contents of CP, NDF, ADF, and WSC are based on standard wet chemistry analytical techniques; however, it is unsuitable for a large number of samples due to its high costly, time-consuming, laborious, and produces pollution in Italian ryegrass (Kong et al., 2005; Wittkop, Snowdon & Friedt, 2012).

Interestingly, a new technique based on the spectroscopy model is aimed at facilitating screening for phenotyping traits for higher growth performance and yield (Araus & Cairns, 2013; Cabrera-Bosquet et al., 2012). As a low cost, rapidity, high-precision and high-throughput technique, near-infrared spectroscopy (NIRS) could predict contents of organic constituents by combining laboratory data and the spectral information (Ramirez et al., 2015; Williams & Norris, 1987b; Wu et al., 2015). The absorbance is measured by different molecular bonds at specific wavelengths, principally C–H, O–H and N–H, which are the basic components of organic compounds of plant tissues (Bokobza, 2002). NIRS is widely utilized for the evaluation of forage quality, including the content of nitrogen, moisture, fiber, structural carbohydrates, amino acids and minerals (Andres et al., 2005; Campo et al., 2013; Cozzolino, Fassio & Gimenez, 2001; Dreccer, Barnes & Meder, 2014; Font, Mdel & Ade, 2006; Fontaine, Hörr & Schirmer, 2001; Meng et al., 2015). The content of CP, NDF and ADF had been accurately predicted in *Oryza sativa* (Kong et al., 2005), *Leymus chinensis* (Chen et al., 2015), *Elymus glabriflorus* (Rushing et al., 2016), *Brassica napus* (Wittkop, Snowdon & Friedt, 2012), and *Salix caroliniana* (Lavin et al., 2016) by the NIRS technique. Besides, NIRS had been also successfully used to quantify the content of WSC in *Triticum aestivum* (Dreccer, Barnes & Meder, 2014), to estimate the phenolic content in *Zea mays* (Meng et al., 2015), and in the screening of early-generation material in cereal breeding programmes (Osborne, 2006). In a word, it had been widely reported that NIRS would be an efficient analytical technique for the rapid prediction of chemical compositions for screening different cereal crop species and forage grasses (Kong et al., 2005). In addition, NIRS does not need solvents or reagent, avoids environment pollution and is regarded as an eco-friendly method, which is accordance with the principles of green chemistry (Cayuela & García, 2017). However, there is little reported study on the development of NIRS calibration models for predicting the forage quality in tetraploid Italian ryegrass populations and the establishment of NIRS calibration models for analysis the CP, NDF, ADF, and WSC contents.

In this study, the main objective was to characterize the methods for measuring the content of CP, NDF, ADF and WSC in a large population of Italian ryegrass cultivars and breeding lines by conventional standard wet chemical analytical techniques and recent near-infrared spectroscopy analyses. Based on the data obtained from two different methods, the partial least squares regression (PLS) would be used for constructing the calibration models of NIRS and the validation of the application potential in Italian ryegrass breeding programs. The optimized NIRS model will quantitatively analyze forage quality parameters of Italian ryegrass in low cost and high throughput ways to further facilitate the speed of the breeding for improving the forage quality.

## Materials and methods

### Materials

A total of 403 Italian ryegrass samples were collected from 34 accessions (15 cultivars and 19 breeding lines, Table 1) at different forage development stage and different locations (Ya’an and Chengdu) from 2014 to 2016. For each sample, a bulked strategy was applied. The fresh samples were inactivated at 105 °C for 30 min, and then oven dried at 65 °C to a constant weight. Finally, the dried samples were ground into powders through a 2 mm sieve and stored in a dry container until use to analysis CP, fiber fractions (NDF and ADF) and WSC.

**Table 1:** The information of Italian ryegrass in this study.

Materials	Origins	Sample number	Materials	Origins	Sample number
Changjiang No.2	Sichuan Agricultural University	24	Changjiang No.2 × Ganxuan No. 1	Sichuan Agricultural University	13
Tetragold	Barenbrug Company	21	Z3	Sichuan Agricultural University	15
Aubade	FF Company	9	Splendor × Ganxuan No. 1	Sichuan Agricultural University	10
Splendor	DLF Company	10	greenland × Ganxuan No. 1	Sichuan Agricultural University	13
Jumbo	Barenbrug Company	9	Splendor × Aubade	Sichuan Agricultural University	11
Chuannong No1.	Sichuan Agricultural University	25	Group B	Sichuan Agricultural University	21
Barwoltra	Barenbrug Company	9	Chenqu × Ganxuan No. 1	Sichuan Agricultural University	11
Diamond T	Clover Group	9	Changjiang No.2 × Tetragold	Sichuan Agricultural University	15
Blue Heaven	Clover Group	10	Jumbo × Ganxuan No. 1	Sichuan Agricultural University	11
Shangnong Tetraploid	Shanghai Jiao Tong University	10	Tetragold × Blue Heaven	Sichuan Agricultural University	22
C8	Sichuan Agricultural University	5	Chenqu × Aubade	Sichuan Agricultural University	6
Abundant	DLF Company	10	Diamond T × Changjiang No.2	Sichuan Agricultural University	6
Jivet	DLF Company	9	Barwoltra × Splendor	Sichuan Agricultural University	4
Group A	Sichuan Agricultural University	16	Barwoltra × liaoyuan	Sichuan Agricultural University	4
Angus No. 1	DLF Company	20	Z4	Sichuan Agricultural University	10
Double Barrel	DLF Company	10	C7	Sichuan Agricultural University	5
Ganxuan No. 1	Jiangxi Livestock Technologies Popularizing Station	10	Aderenalin	Beijin Green Animal Husbandry S&T Development CO.,LTD	10

### Near infrared spectra (NIRS) collection

Near infrared reflectance spectroscopy analysis was performed using a Bruker MPA Fourier Transform near infrared (FT-NIR) spectrophotometer (Bruker, Bremen, Germany), equipped with a quartz beamsplitter and a PbS detector. It was also equipped with an integrating macrosample sphere and a rotating sample cup, allowing the scanning of large areas of the samples. NIR spectra of ground samples were obtained with the following procedure: aliquots of around 25 g of dried samples were placed in rotating sample cup, and scanned on reflectance mode in the spectral range from 4,000 to 12,500 cm^−1^ at room temperature (∼20 °C). In each of the reflectance measurements, 64 scans were run and the resolution used for spectral analysis was 8 cm^−1^. Spectrum were produced with 2,203 date points per sample (Table S1). Background corrections were made before each sample was scanned. Samples were measured in triplicate, which increased the scanned surface of samples for reducing errors. The spectral absorbance values were recorded as log1/R, where R is the sample reflectance.

### Biochemical analysis

The Kennard-Stone algorithm was used to select a subset of 123 samples (out of the 403 samples) for biochemical analysis. This algorithm selects a defined number of representative samples that systematically cover the spectral variation of all samples. Samples from the selected subset were analyzed for CP, NDF, ADF, and WSC by standard wet chemical analytical techniques (Table S1). The data generated from biochemical methods will act as the reference data for future analysis. All experiments were performed with three biological replicates.

### Determination of Crude Protein content

The CP content were determined by wet chemistry analysis according to the Kjeldahl method (AOAC Official Method 984.13.15) (AOAC, 1990) with the Kjeltec™ 8400 analyzer unit (FOSS, Hoganas, Sweden). The ground samples (0.5 g) were added into a 250 ml TKN digestion tube with 10 ml concentrated sulfuric acid and two digestive tablets (Beijing Jinyuanxingke Technology, Beijing, China). Blanks containing all these reagents were simultaneously processed. All tubes were digested in the preheated digestion block (Tecator™ digestor auto; FOSS, Hoganas, Sweden) at 420 °C for 90 min or until the samples were green and clear. The Kjeldahl digests procedure was carried out using a Kjeltec™ 8400 analyzer unit (FOSS, Hoganas, Sweden). The CP content was calculated using the following equation:

\begin{eqnarray*}\text{CP} \left( \text{%}\text{DM} \right) = \frac{ \left( V1-V2 \right) \times C\times 1.4007\times 6.25}{M} \times 100 \end{eqnarray*}

Where: *V*1 = volume (ml) of standard HCl required for sample; *V*2 = volume (ml) of standard HCl required for blank; *C* = molarity of standard HCl; 1.4007 = milliequivalent weight of *N* × 100; 6.25 = average coefficient of nitrogen conversion into proteins; *M* = sample weight in grams.

### Determination of neutral detergent fiber and acid detergent fiber contents

The NDF and ADF contents were measured by using the methods described by Goering & Van Soest (1970) and Van Soest, Robertson & Lewis (1991) with 0.5 g pulverized samples in Automatic Fiber Analyzer (ANKOM 2000 Fiber Analyzer; ANKOM Technology, NY, USA). The contents of NDF and ADF were calculated using the following equation:

\begin{eqnarray*}\text{NDF}  \left( \text{%}\text{DM} \right) & = \frac{ \left( M2- \left( M1\times C1 \right) \right) }{M} \times 100 \end{eqnarray*}

\begin{eqnarray*}\text{ADF}  \left( \text{%}\text{DM} \right) & = \frac{ \left( M3- \left( M1\times C1 \right) \right) }{M} \times 100 \end{eqnarray*}

Where: *M* = Sample weight; *M*1 = Bag tare weight; *M*2 = Weight of organic matter after extraction by neutral detergent; *M*3 = Weight of organic matter after extraction by acid detergent; *C*1 = Ash-corrected blank bag factor (a running average of the loss of weight after extraction of the blank bag/original blank bag); *C*2 = Ash-corrected blank bag factor (a running average of the loss of weight after extraction of the blank bag/original blank bag).

### Determination of water soluble carbohydrates content

The WSC content were determined as follows: 0.1 g dried samples were ground with 1 ml distilled water, and the homogenate were transferred to a 2 ml tubes and then water bath for 10 min at 95 °C. After centrifugation at 8,000× g for 10 min at 25 °C, all supernatants were collected and the final volume was adjusted to 10 ml with distilled water. The content of WSC was detected by using assay kits (Suzhou Comin Biotechnology Co., Ltd, Suzhou, China). Briefly, 40 µl supernatants were put in a 1.5 ml eppendorf tube with 40 µl distilled water, 20 µl mix solution and 200 µl concentrated sulfuric acid. The reaction mixture was shaken and incubated in a water bath for 10 min at 95 °C and then cooled to room temperature. A total of 200 µL reaction mixture was transfered to a 96 well EIA/RIA plate and read the absorbance at 620 nm using a Thermo Scientific Multiskan™ Go (Thermo Scientific, Waltham, MA, USA).

The contents of WSC were calculated as follows:

\begin{eqnarray*}\text{WSC}  \left( \text{%}\text{DM} \right) = \frac{2.34\times \left( \Delta A+0.07 \right) }{W\times 10} \end{eqnarray*}

Where: Δ*A* = The absorbance of test tube; *W* = Sample weight.

### Calibration and validation of near infrared spectra models

All 123 selected samples were used to construct the near infrared spectra models in the OPUS software (Bruker, version 5.5) by using partial least square (PLS) regression throughout the process to calculate the correlation between spectral data and laboratory data. The raw spectral data were transformed by several pretreatments before the calibration process, including standard normal variate (SNV), standard multiple scatter correction (MSC), minimum-maximum normalization (MMN), first derivative (FD), second derivative (SED), straight line subtraction (SLS), constant offset elimination (COE), and a combination of FD with MSC, SLS, SNV to remove artifacts and imperfections from the date. For the validation of the models, all 123 samples were split in 93 and 30 for cross validation and external validation sets, respectively, according to the range of the chemical values. Cross-validation is conducted when developing NIRS models by using PLS regression, which attempted to screen the optimal ranks and avoid over-fitting of the data (Shenk & Westerhaus, 1991). Furthermore, external validation subsets were applied to evaluate and validate the potential accuracy of the models.

The statistical methods applied in this study included the coefficient of determination calculated in cross-validation (*R*^2^_CV_) and external validation (*R*^2^_V_), the root mean square error of calibration (RMSEC), the root mean square error of cross-validation (RMSECV), and the root mean square error of prediction (RMSEP). Moreover, the ratio of prediction to deviation (RPD), which indicated the correlations between the SD of the standard wet chemical analyzed data and prediction data by NIRS model (RMSECV or RMSEP) (Williams & Sobering, 1996), was applied to estimate the prediction ability of the model. Besides, the prediction error relative to laboratory (PRL) and the range error ratio (RER, calculated as the ratio between the range of standard wet chemistry values and the RMSECV or the RMSEP of the parameters) were calculated in this study, and were considered as an additional criteria for determined the prediction ability of each of the models.

## Result

### Chemical characteristics of forage quality attributes

**Figure 1:**
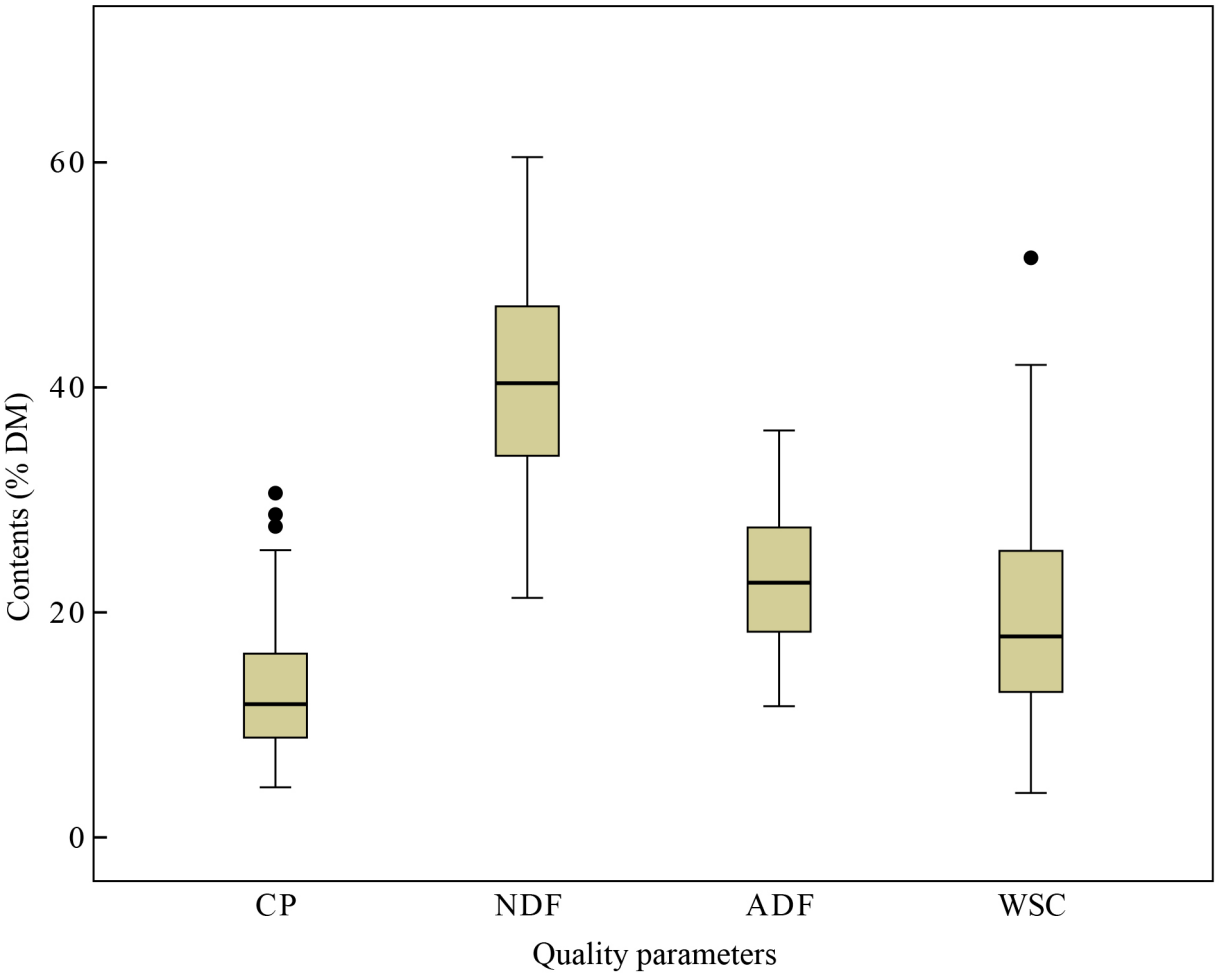
Quality parameters content. Box and whisker diagrams of the reference values (i.e., values obtained using conventional wet chemistry) measured for quality parameters content (%DM), including crude protein (CP), acid detergent fiber (ADF), neutral detergent fiber (NDF) and water soluble carbohydrate (WSC) in Italian ryegrass. Box plots show median values (solid horizontal lines), 50th percentile values of the data range (box outlines) and whiskers 100th percentile values of data (whiskers), with the exception of the outliers shown as individual points.

The content of CP, NDF, ADF and WSC were detected by standard wet chemical analytical techniques in laboratory and the CP expressed in %DM ranged from 4.45 to 30.6, NDF ranged from 21.29 to 60.47, ADF ranged from 11.66 to 36.17, and WSC ranged from 3.95 to 51.52% in %DM, respectively (Fig. 1). The variability of NDF and WSC contents were observed highest (±9.35 and ±9.40 %DM), while CP content was lowest (±5.71 %DM). Besides, the calibration (93 samples) and the validation (30 samples) data sets were also analyzed separately, including the values between maximum and minimum values, mean, and SD (Table 2). The variation of the CP, NDF, ADF and WSC content could be considered acceptable and broad enough for development of the aimed NIRS calibration models.

### Features of NIRS spectra

**Figure 2:**
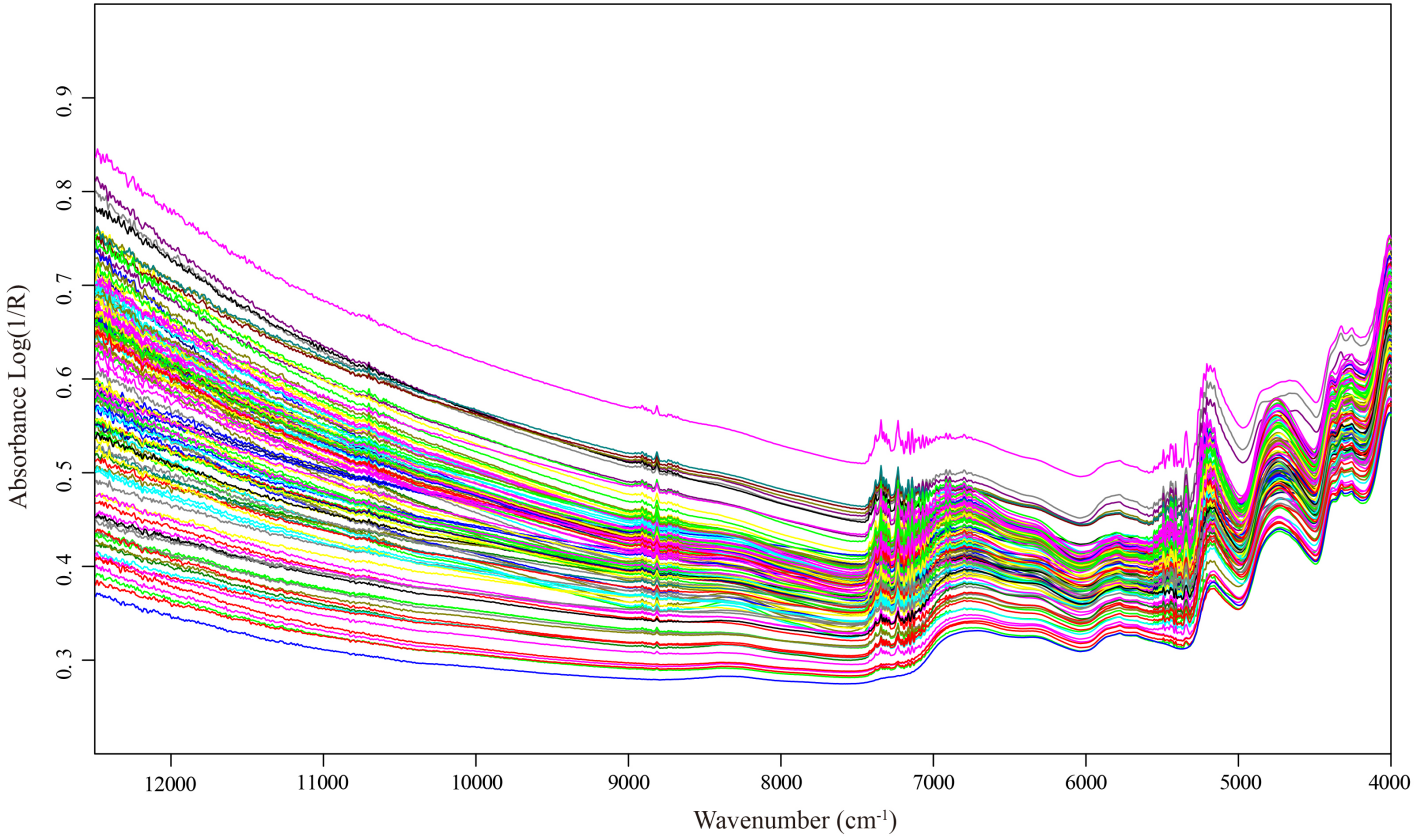
NIR spectra of each Italian ryegrass sample. NIR spectra of each Italian ryegrass sample in the wavelengths range of 4,000–12,500 cm^−1^.

The raw spectral data of 123 selected samples exhibited the general spectral features that one expects from dried plant samples (Fig. 2). It is obviously shown that peaks and valleys were presented in the spectra, which indicated the different chemical component characteristics of Italian ryegrass samples. In the wavenumber region 4,000–12,500 cm^−1^, there were five main absorption peaks located at wavelength approximately 4,240, 4,740, 5,170, 5,800, and 6,800 cm^−1^, respectively. After comparison to the origin of near-infrared absorption bands, we found that these wavelength related to critical functional groups as carbon atoms and hydrogen (C–H), hydrogen atoms and oxygen (O–H) and ammonia (N–H) in CP, NDF, ADF and WSC.

**Table 2:** Summary statistics calibration and validation sets of CP, NDF, ADF and WSC contents (%DM) analyzed by standard wet chemistry methods in Italian ryegrass from the calibration and the validation sets.

Parameter	Calibration					Validation				
	*N*	Mean	SD	Min	Max	*N*	Mean	SD	Min	Max
CP	93	12.94	5.82	4.45	30.60	30	12.80	5.36	5.10	25.53
NDF	93	40.47	9.48	21.29	60.47	30	40.48	8.95	24.14	55.41
ADF	93	22.92	6.12	11.66	36.17	30	22.92	5.81	13.23	33.88
WSC	93	19.93	9.63	3.95	51.52	30	19.65	8.68	5.33	39.90

**Notes.**

*N* number of samples SD standard deviation Min minimum value Max maximum value

### NIRS model calibrations and cross validation

**Figure 3:**
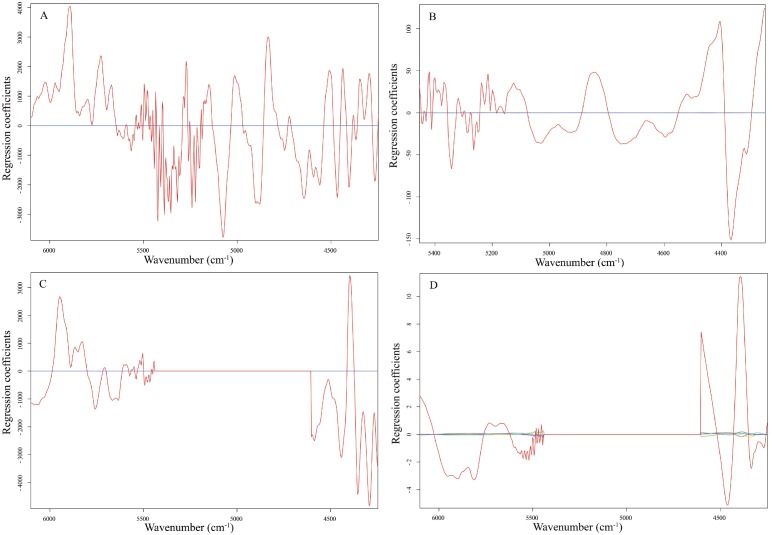
Regression coefficients of the PLS models. Regression coefficients of the PLS models for CP (A), NDF (B), ADF (C), and WSC (D).

The PLS regression of NIRS spectra and laboratory values constructed good calibration models for CP, NDF, ADF and WSC content of Italian ryegrass with the cross-validation processing simultaneously. When a wide range of possible combinations of different spectral pretreatments were tested, the optimal combinations with lowest RMSECV values were applied to construct the models. The results showed that FD+MSC combinations could well improve the linearity relation between reference and spectral values of CP, the SNV pretreatment improved the linearity relation of NDF, FD improved the linearity relation of ADF, and MMN improved the linearity relation of WSC (Table 3). Besides, we also got the optimal wavenumber based on these spectral pretreatments, including the calibration model for CP was developed at 4,247–6,102 cm^−1^, NDF at 4,247–5,450 cm^−1^, ADF at both 5,446–6,102 cm^−1^ and 4,247–4,602 cm^−1^ wavenumber, and the WSC was same as ADF (Table 3). The regression coefficients contributing for the CP, NDF, ADF and WSC models are shown in Fig. 3. In these graphs, wavelengths within the horizontal line have zero contribution to the models. The coefficients for CP model presented abundant spectral variable within the optimal wavenumber regions, which provide efficient contribution in the calibration process. The coefficients for NDF, ADF, and WSC models showed an obvious positive contribution at the peak of 4,405 cm^−1^, which indicated that the distinct spectral region is possibly related to a C–H+O–H combination band attributed to cellulose and sugar.

**Table 3:** Cross-validation statistics of NIRS calibrations for the estimation of CP, NDF, ADF and WSC contents (%DM) in Italian ryegrass obtained by PLS regression.

Parameter	SCM	Spectrum range (cm^−1^)	Ranks	*R*^2^_CV_	RMSECV	RPD_C_	SEL_C_	PRL_C_	RER
CP	FD +MSC	4,247–6,102	10	0.99	0.68	8.58	1.56	0.43	38.57
NDF	MSC	4,247–5,450	7	0.94	2.23	4.25	1.62	1.38	17.57
ADF	FD	4,247–4,602; 5,446–6,102	6	0.92	1.68	3.64	1.43	1.17	14.59
WSC	MMN	4,247–4,602; 5,446–6,102	7	0.88	3.11	3.10	1.66	1.88	15.30

**Notes.**

SCM scatter correction methods Ranks number of principal component used for calibration *R*^2^_CV_ determination coefficient of cross-validation RMSECV root mean square error of cross-validation RPD_C_ ratio of prediction to deviation for the calibration (SD/RMSECV) SEL_C_ standard error of laboratory in calibration PRL_C_ prediction error relative to laboratory of calibration models RER_CV_ range error ratio for the calibration models (max–min)/RMSECV

Based on the constructed optimal model for each trait, a comparison of four models was conducted. The results showed that the best model with lowest RMSECV and the highest *R*^2^_CV_ values (RMSECV = 0.68, *R*^2^_CV_ = 0.99) was the CP calibration model, while the highest RMSECV and the lowest *R*^2^_CV_ value was obtained from the WSC calibration model, indicating that the NIRS model for predicting CP content was well suitable in Italian ryegrass (Table 3). Correspondingly, the PRL_C_ (RMSECV/SEL_C_) ratio scales the adjusted prediction error (RMSECV) relative to the precision of the standard wet chemistry method (SEL_C_). For calibrations approaching laboratory precision in this study, the value of PRL_C_ ranged from 0.43 (in CP model) to 1.88 (in WSC model), which the prediction errors all located whin 2 times of the standard wet chemistry precision, indicating that four models were sufficient for application. The RPD_C_ (SD/RMSECV) ratios ranged from 3.10 for WSC to 8.58 for CP, which represented the relationship between the natural variation of the calibration population and the prediction errors of the calibration model (Table 3). Furthermore, the value of RER were calculated in the calibration models and all the values were higher than the recommended (RER > 10) for screening purposes.

### External validation of NIRS calibrations models

**Figure 4:**
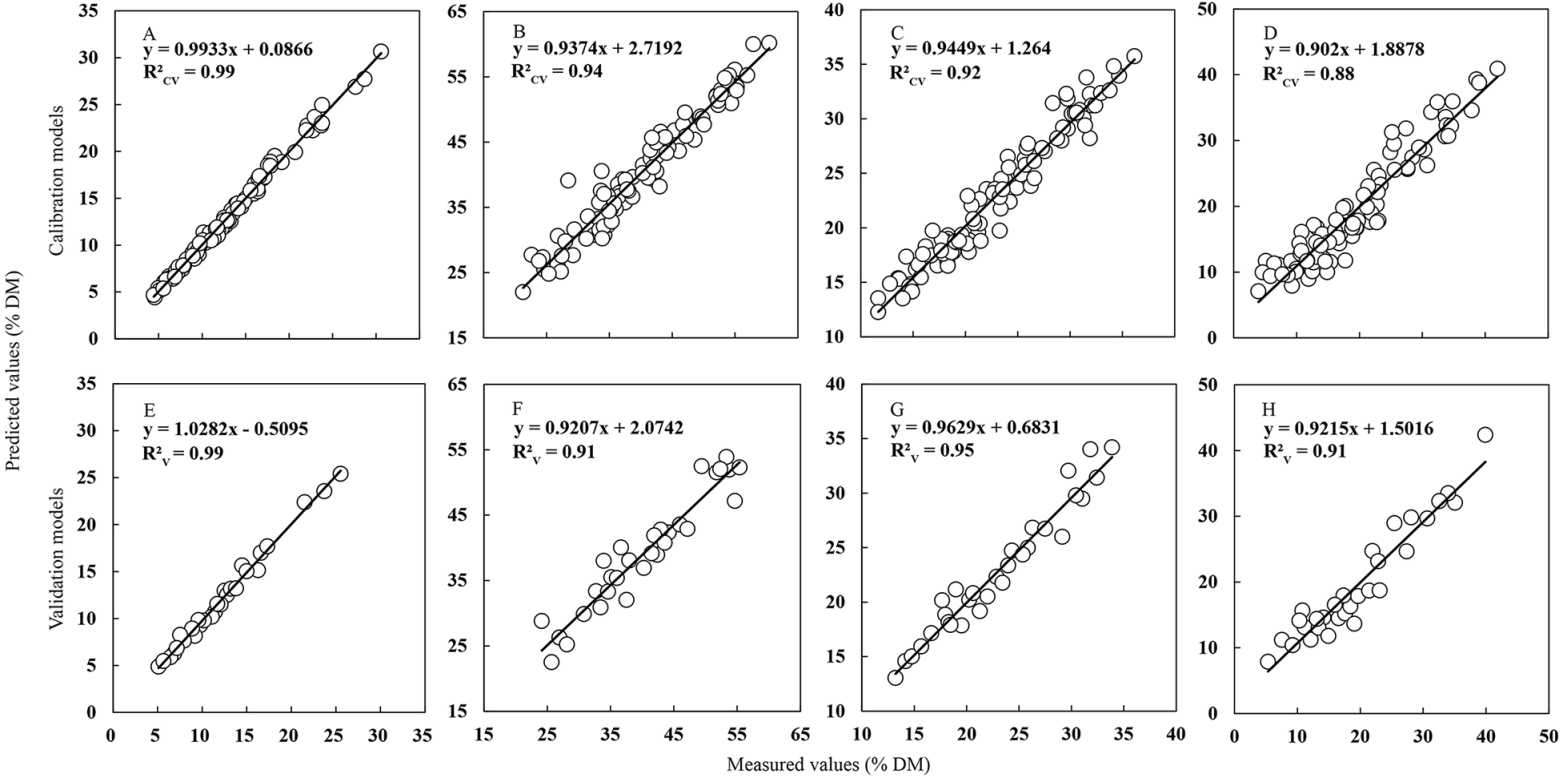
Relationship between values measured with the standard reference methods and values predicted by NIRS. Relationship between values measured with the standard wet chemistry methods (*x* axis) and values predicted by NIRS (*y* axis) in the calibration and external validation sets for CP (A, E), NDF (B, F), ADF (C, G) and WSC (D, H) in Italian ryegrass. The solid line is the relationship between measured and predicted values in the calibration and validation sets for quality parameters.

External validation of the NIRS calibrations models was carried out using the validation data subset (including 30 samples), which had similarly broad distribution of CP, NDF, ADF and WSC content with the calibration data set (Table 4 and Fig. 4). For all individual parameters, robust and parsimonious calibration models were identified and their predictive ability in the external validation was retained. Corresponding to the results of calibration model, the value of the determination coefficient for CP content in external validation model still kept the highest (*R*^2^_V_ = 0.99) compared to other parameters, which revealed the powerful prediction ability of the NIRS model for CP content in Italian ryegrass. All *R*^2^_V_ values of ADF, NDF and WSC model reached the acceptable thresholds of 0.90, which efficiently validated the reliability of the models for these parameters. On the other hand, the calculated RPD_P_ values ranged from 9.37 for CP to 3.39 for WSC, and the value of PRL ranged from 1.56 for WSC to 0.41 for CP, which could well reflect the accuracy of the models. Additionally, all the RER values (ranged from 12.03 to 35.72) in this study exceeded the recommended threshold values for screening purposes. In total, all results characterized before in external validation proved that the NIRS models constructed had an excellent quantitative ability and a powerful prediction for further Italian ryegrass forage quality evaluation in the field, especially for the CP content.

**Table 4:** External validation statistics obtained from regression equations of laboratory values of CP, NDF, ADF and WSC contents (% DM) in Italian ryegrass and NIRS predicted values for the validation set.

Parameter	*R*^2^_V_	RMSEP	RPD_P_	SEL_V_	PRL_P_	RER_P_
CP	0.99	0.57	9.37	1.41	0.41	35.72
NDF	0.91	2.60	3.44	1.77	1.47	12.03
ADF	0.95	1.32	4.40	1.29	1.02	15.64
WSC	0.91	2.56	3.39	1.64	1.56	13.50

**Notes.**

*R*^2^_V_ coefficient of determination of prediction models RMSEP root mean square error of prediction RPD_P_ ratio of prediction to deviation for the prediction models (SD/RMSEP) SEL_V_ standard error of laboratory in validation PRL_P_ prediction error relative to laboratory of prediction models RER_P_ range error ratio for the prediction models (max–min)/RMSEP

## Discussion

For reliable selection of a large number of accessions with improved traits, an analytical method with good precision and a high-throughput is required for forage quality evaluation. Owing that the NIRS technique effectively combines laboratory value and the spectral information, it has been regarded as a new fast and reliable method compared to traditional analytical methods. Recently, it had been reported that the NIRS technique could be successfully applied for the screening of forage quality parameters in *Leymus chinensis* (Chen et al., 2015), *Elymus glabriflorus* (Rushing et al., 2016), *Glycine max* (Asekova, 2016), *Oryza sativa* (Kong et al., 2005), *Brassica napus* (Wittkop, Snowdon & Friedt, 2012), and *Sorghum bicolor* (Fox et al., 2012). In this study, the most critical quality parameters comprising CP, NDF, ADF and WSC contents were detected in 34 Italian ryegrass accessions at different development stage and the results showed that the range of most parameters was broader than the range in previous studies (Asekova, 2016; Fox et al., 2012; Williams & Norris, 1987a), which represented a wider application of our NIRS model constructed based on these data.

The reflectance spectrum is the result of the absorption features of a sample for each chemical composition. Different chemical bonds absorb at different wavelengths, the interactions among chemical components, and the differences in particle size, shape and orientation produced the multiple absorption bands in the raw spectral data (Williams & Norris, 1987a). Hence, the raw spectral data contain a large amount of information. Wu et al. (2015) obtained the key wavelengths of 1,180–2,492, 408–2,492, and 1,180–2,492 nm from optimal models of cellulose, hemicelluloses, and lignin in sweet sorghum, respectively. Shi & Yu (2017) suggested that the key wavelengths of CP content in wheat was 1,800–2,300 nm. The critical wavelengths identified in this study were located in 4,247–6,102 and 4,247–5,450 cm^−1^ for CP and NDF content, and both wavelengths 5,446–6102 and 4,247–4,602 cm^−1^ could for ADF and WSC.

Calculating the spectral frequencies constituted by optimal wavelengths corresponds to identifying the key molecular bond regions located in the spectrum, contributing to the well relations between spectrum data and the contents of chemical composition (Curran, 1989). The model constructed within these regions will produce the minimum errors when conduct the quantitative determinations and qualitative analysis (Workman Jr & Weyer, 2012; Xiaobo et al., 2010). Overtones vibrations and combinations vibrations of the functional groups like C–H, O–H and N–H always produce overlapping absorptions (Chung & Potma, 2013; Siesler et al., 2008). The optimal wavelengths identified in this study were mainly correlated to the stretching and bending of the chemical bonds between C–H, O–H, N–H, and C–O, which indicated that the organic chemical compounds absorbed in these wavenumbers are mainly cellulose, sugar and starch, and lignin (Curran, 1989; Decruyenaere et al., 2012; Workman Jr & Weyer, 2012).

In general, the calibrations obtained based on standard wet chemistry values and the spectral data for the chemical compositions had good prediction ability and reproducibility (Williams, 2001; Williams, 2007). CP content may be the most commonly measured variable in forage and feedstuffs. Previous studies have reported that CP concentrations could be well quantified by NIRS in forage (Andueza et al., 2011; Chen et al., 2015; Jafari et al., 2003; Rushing et al., 2016). Consistently, the coefficient of determination and RPD values associated with the CP model obtained in this study were quite satisfactory (with *R*^2^_V_ value of 0.99 and RPD value of 9.37), according to a guideline scale suggested by Malley, Martin & Ben-Dor (2004) that a NIRS equations model was considered excellent for screening if the *R*^2^_V_ > 0.95 and RPD > 4, successful if *R*^2^_V_ = 0.9 − 0.95 and RPD = 3–4, and the model inadequate if *R*^2^_V_ < 0.7 and RPD < 1.75. The values which were higher than in the other forages included *Leymus chinensis* (*R*^2^_V_ = 0.91, RPD = 3.20) (Chen et al., 2015), *Elymus glabriflorus* (*R*^2^_V_ = 0.97, RPD = 5.37) (Rushing et al., 2016), and *Glycine max* (*R*^2^_V_ = 0.91, RPD = 3.25) (Asekova, 2016).

In order to further evaluate the accuracy of the models, the further statistics (PRL ratio and RER value) need to be considered. The PRL (RMSECV or RMSEP/SEL) ratio scales the prediction error relative to the precision of the standard wet chemistry methods. Some reports suggested that the RMSEP should range within two times of the SEL which indicates the adequate of the developed NIRS calibrations model (Kong et al., 2005; Velasco & Möllers, 1998; ZumFelde et al., 2007). The ratios of the model for CP content observed in this study was 0.41, which falls within the recommended range, indicating that the developed calibration in this study has a good precision and is suitable for accurate routine use (Windham, Mertens & Barton, 1989). The RER value obtained in the CP model was 35.72, which was far greater than the recommended threshold value of RER > 20 (Windham, Mertens & Barton, 1989). However, Williams & Norris (1987a) suggested that RER > 10 could indicate high utility of the model while limited practical utility when 3 < RER < 10. In conclusion, the CP model has a stable performance and a better predictive power compared to others, and can be widely used to evaluate the forage quality of Italian ryegrass and applied in rumination feed in future.

NDF and ADF contents always effected the digestion of forage for the livestock, which were considered as two important limited factors for the estimation of the nutritive qualities of feed and forage (Wolfrum & Lorenz, 2009). A large number of studies showed that NDF and ADF concentrations could be well predicted by NIRS in forage (Kong et al., 2005; Stubbs, Kennedy & Fortuna, 2010; Wittkop, Snowdon & Friedt, 2012; Chen et al., 2015; Rushing et al., 2016). In *Leymus chinensis*, Chen et al. (2015) constructed NIRS models by using partial least squares regression, multiple-linear regression and principal component regression, which successfully predicted the contents of NDF and ADF, but the NIRS model for NDF are less accurate than those for ADF. The similarity results were found in *Oryza sativa* (Kong et al., 2005) and *Brassica napus* (Wittkop, Snowdon & Friedt, 2012). In this study, the performance of the models for NDF (*R*^2^_V_ = 0.91, RPD = 3.44) and ADF (*R*^2^_V_ = 0.95, RPD = 4.40) were successful and the precision of the models were consistent with previous studies. Although the precision of the NDF model was also lower compared to the ADF, it was still sufficient to differentiate between high (60.47 %DM) and low (21.29 %DM) NDF content in Italian ryegrass. Interestingly, the coefficient of determination in the calibration process (*R*^2^_CV_ = 0.94) was higher than the validation (*R*^2^_CV_ = 0.91), which could be contributed to the fact that the whole set of samples employed are not evenly distributed in terms of composition (Keim, Charles & Alomar, 2015). Murray (1988) suggested that a suitable set of samples for NIRS analysis should be wide and evenly distributed in terms of their composition, and the extreme values will be less represented when separating the sample set to calibration and validation subset. Besides, the NIRS model of WSC content also had a good performance (*R*^2^_V_ = 0.91 and RPD = 3.39) for estimation in application, which is consistent with previous studies that estimated carbohydrate content in foliar tissues using NIRS (Chen et al., 2014; Quentin et al., 2017; Ramirez et al., 2015).

Overall, NIRS offered enormous flexibility for analyzing multiple constituents of plant tissues (Marten et al., 1984). Moreover, the success of the NIRS method also results from the time saving and costs associated with the analysis. Indeed, we saved about 80% of normal laboratory costs by using the NIRS method than the standard wet chemistry. Finally, NIRS analysis produced no chemical wastes, which should be an important incentive for environmental friendliness and for reducing the cost of the reagents and waste disposal.

## Conclusions

The prediction of forage quality parameters by the NIRS model is a relatively inexpensive, rapid, reliable and eco-friendly method compared to the standard wet chemistry methods, requiring a relatively small quantity of samples and predicts several concentrations of components simultaneously. In this study, we developed four optimal NIRS models to predict the CP, NDF, ADF, and WSC contents in Italian ryegrass samples. A broader range of CP, NDF, ADF and WSC contents (%DM) was detected (4.45–30.60, 21.29–60.47, 11.66–36.17 and 3.95–51.52, respectively) by standard wet chemistry methods for developing NIRS models. The optimal models were developed based on the laboratory data and the spectral information by partial least squares regression in the key wavelengths (4,247–6,102 and 4,247–5,450 cm^−1^ for CP and NDF, both wavelengths 5,446–6,102 and 4,247–4,602 cm^−1^ for ADF and WSC). The models were validated using a completely independent set of samples and have relatively high coefficients of determination (${R}_{\mathrm{V }}^{2}$, CP = 0.99, NDF = 0.91, ADF = 0.95, WSC = 0.91) and ratio of prediction to deviation (RPD, CP = 9.37, NDF = 3.44, ADF = 4.40, WSC = 3.39). In conclusion, the result suggested that routine screening for forage quality parameters with large numbers of samples is available with the NIRS model in Italian ryegrass breeding programs, as well as facilitating graziers to monitor nutritional dynamic in the forage development stage and to identify the optimal utilization period of forage grasses.

##  Supplemental Information

10.7717/peerj.3867/supp-1 Table S1 The chemical values and NIR spectral data of 123 samples

## Additional Information and Declarations

### Data Availability

The following information was supplied regarding data availability: The raw data has been provided as a Supplemental File.
